# Using Machine Learning Methods Incorporating Individual Reader Annotations to Classify Paediatric Chest Radiographs in Epidemiological Studies

**DOI:** 10.12688/wellcomeopenres.17164.1

**Published:** 2021-11-12

**Authors:** Paul Mwaniki, Timothy Kamanu, Samuel Akech, M. J. C Eijkemans

**Affiliations:** 1 Kenya Medical Research Institutes - Wellcome Trust Research Programme, Nairobi, Kenya; 2 School of Mathematics, University of Nairobi, Nairobi, Kenya; 3 ulius Center for Health Sciences and Primary Care, Department of Data Science and Biostatistics, University Medical Center Utrecht, Utrecht University, Utrecht, The Netherlands

**Keywords:** Machine learning, Chest Radiograph, Pneumonia

## Abstract

**Introduction**: Epidemiological studies that involve interpretation of chest radiographs (CXRs) suffer from inter-reader and intra-reader variability. Inter-reader and intra-reader variability hinder comparison of results from different studies or centres, which negatively affects efforts to track the burden of chest diseases or evaluate the efficacy of interventions such as vaccines. This study explores machine learning models that could standardize interpretation of CXR across studies and the utility of incorporating individual reader annotations when training models using CXR data sets annotated by multiple readers.

**Methods**: Convolutional neural networks were used to classify CXRs from seven low to middle-income countries into five categories according to the World Health Organization's standardized methodology for interpreting paediatric CXRs. We compared models trained to predict the final/aggregate classification with models trained to predict how each reader would classify an image and then aggregate predictions for all readers using unweighted mean.

**Results**: Incorporating individual reader's annotations during model training improved classification accuracy by 3.4% (multi-class accuracy 61% vs 59%). Model accuracy was higher for children above 12 months of age (68% vs 58%). The accuracy of the models in different countries ranged between 45% and 71%.

**Conclusions**: Machine learning models can annotate CXRs in epidemiological studies reducing inter-reader and intra-reader variability. In addition, incorporating individual reader annotations can improve the performance of machine learning models trained using CXRs annotated by multiple readers.

## Introduction

Chest radiograph (CXR) is an essential tool in the diagnosis of conditions affecting the lungs. CXR can improve the specificity of pneumonia diagnosis, given that clinical diagnosis is sensitive but non-specific ( Cardoso *et al*., 2010; Scott *et al*., 2012). Interpretation of CXR by clinicians for diagnosing pneumonia is subjective, making the comparison of results from different studies or periods difficult to interpret ( Ben Shimol *et al*., 2012; Levinsky *et al*., 2013; Williams *et al*., 2013). The World Health Organization (WHO) developed a standardized methodology for interpreting paediatric CXR for categorization of radiological pneumonia to enable consistent assessment of burden of pneumonia and impact of interventions such as vaccines ( WHO, 2001). During the assessment of the developed tool, it was noted that while there was no variation in interpretation of CXR between radiologists and clinicians, readers from different sites had varying levels of sensitivity and specificity. Readers from two sites had low sensitivity but high specificity, while those from a third site had high sensitivity but low specificity ( Cherian *et al*., 2005). Fancourt (2017a) observed that agreement between primary readers declined between the first and second phases of annotation, suggesting that intra-reader variability may also be of concern. Inter-reader variability in the interpretation of CXRs has also been observed in the diagnosis of adult pneumonia and tuberculosis ( Melbye & Dale, 1992; Yerushalmy, 1969).

Recent developments in machine learning and computer vision have shown that machine learning models can be as good as radiologists and clinicians at interpreting CXRs ( Lakhani & Sundaram, 2017; Rajpurkar *et al*., 2018; Rajpurkar *et al*., 2017). In addition, machine learning models can reduce variability in CXR interpretation across multiple sites or studies if the models are generalizable across sites/studies. Machine learning models may also be appropriate in epidemiological studies that require interpretation of large numbers of CXRs.

Machine learning models for classifying medical images are trained to predict the final classification of a given image, obtained by aggregating annotation from multiple human readers ( Rajpurkar *et al*., 2018). While aggregated annotations are likely to have less misclassification noise, there might be additional training signals in each reader's annotation that may be lost by aggregating. Therefore, we propose an alternative approach where the models are trained to classify how each reader would classify a given image and then aggregating the predictions for all readers. Combining predictions for multiple readers is similar to ensemble methods in machine learning, where predictions from multiple models are averaged. On average, the performance of model ensembles is expected to be at least as good as the best single model ( Goodfellow *et al*., 2016). However, unlike ensemble models where multiple models are trained, we train a single model that takes a CXR image and reader identifier as inputs and produces a prediction on how that reader would have classified the image.

This study compares the classification performance of models trained to predict the final/aggregate classification with models trained to predict how each reader would classify a given image and then aggregating the predictions of all readers. The models are trained to classify the Pneumonia Etiology Research for Child Health (PERCH) data-set that contains CXR images of paediatric patients hospitalized with pneumonia ( Fancourt *et al*., 2017b; Fancourt *et al*., 2017a).

## Methods

### Ethics approval

The study protocol for the initial PERCH study was approved by the Institutional Review Boards or Ethical Review Committees for each of the seven institutions and at The Johns Hopkins School of Public Health. Parents or guardians of participants provided written informed consent. We made a data request for secondary data analysis to John Hopkins School of Public Health. 

### Data

The PERCH study data-set consists of 4,172 CXRs from 4,008 paediatric patients hospitalized with severe or very severe pneumonia (WHO pneumonia classification). PERCH aimed at studying pneumonia aetiology in children and was conducted in nine sites from seven low and middle-income countries: Kilifi, Kenya; Basse, The Gambia; Nakhon Phanom and Sa Kaeo, Thailand; Bamako, Mali; Soweto, South Africa; Lusaka, Zambia; and Dhaka and Matlab, Bangladesh. The CXR images were classified into five categories based on WHO standardized classification of paediatric CXR: consolidation; other infiltrate; both consolidation and other infiltrate; normal or uninterpretable ( Cherian *et al*., 2005). Digital CXR imaging machines were used to acquire images in all sites except Zambia and Matlab, where analogue machines were used, and the films were then scanned into digital format. More than 98% of the CXR were taken anterior-posterior (AP).

There were 18 readers, 14 initial readers (nine paediatricians and five radiologists) and four arbitrators (radiologists). The initial readers consisted of two readers from each country who received training on the WHO methodology from the arbitrators. Whenever the two initial readers gave conflicting interpretations, two arbitrator readers with extensive WHO methodology experience were randomly chosen to review the image. If the two arbitrators still came to conflicting interpretations, the two arbitrators held a consensus discussion to make a final decision. Finally, the arbitrators reviewed 10% of images with initial concordance for quality control ( Fancourt *et al*., 2017b).

**Figure 1.**
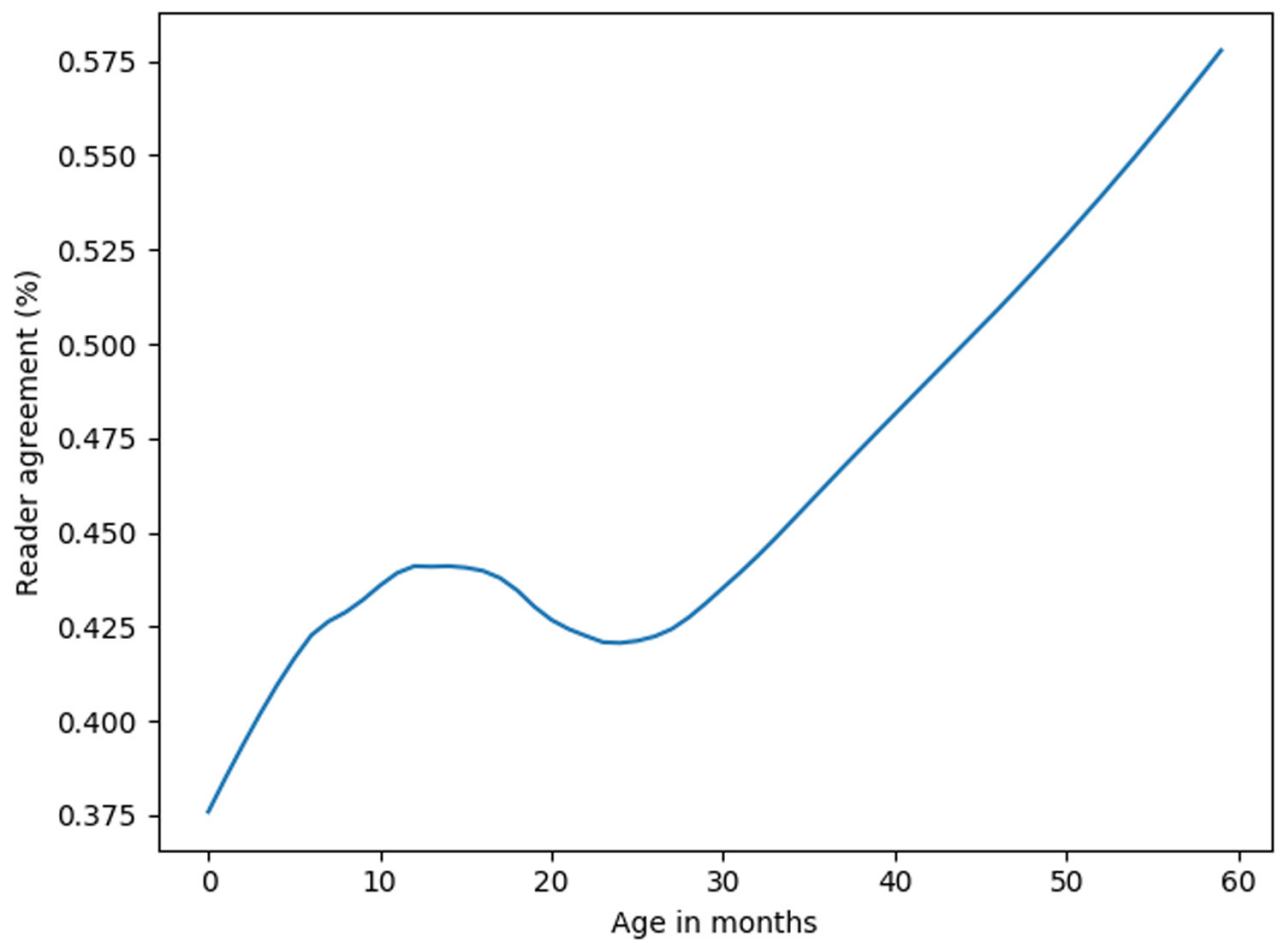
Lowess curve: Agreement of first and second reader by age. The agreement between first and second human readers improved with increase in children's age.

**Figure 2.**
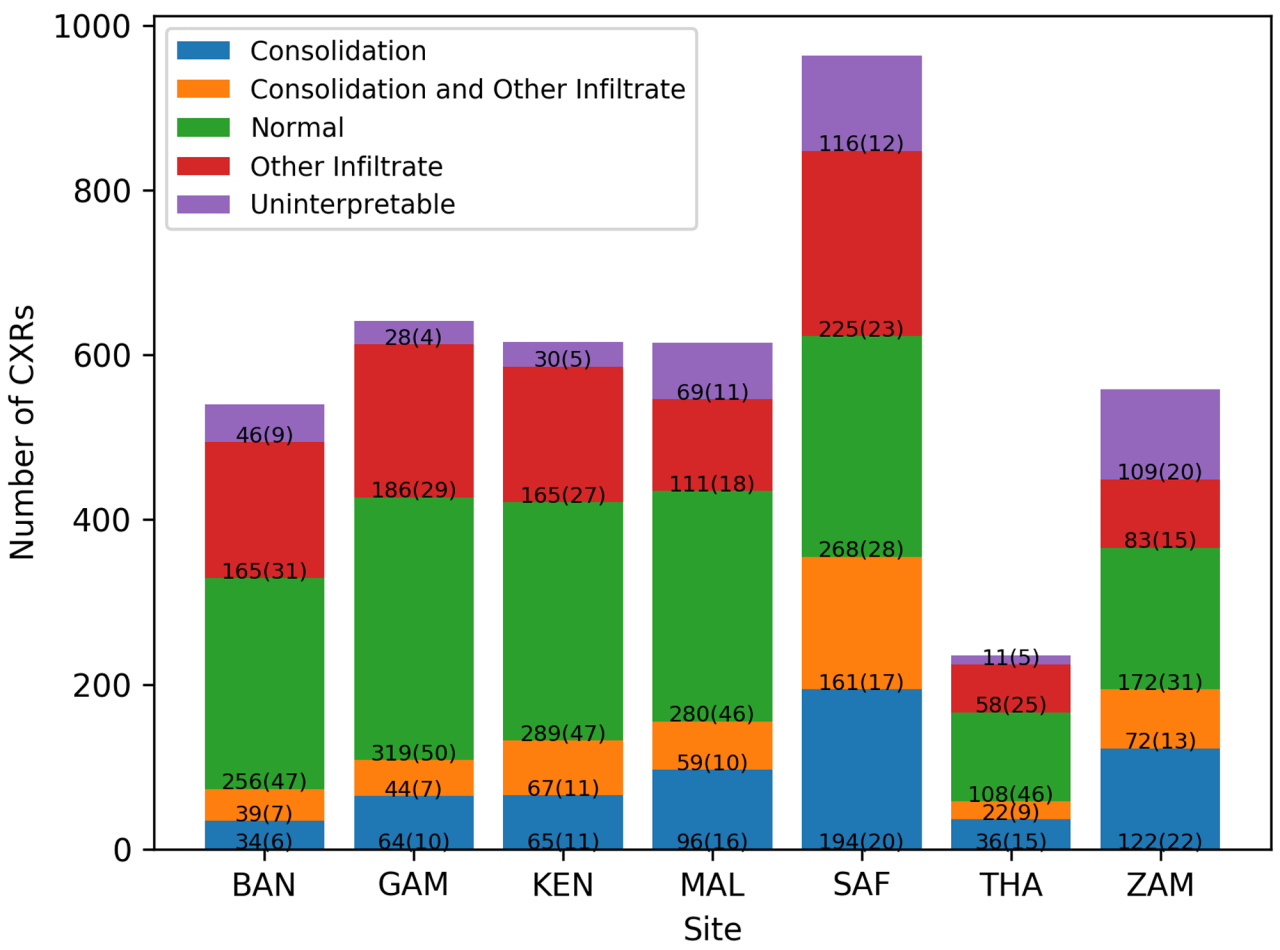
Number (percentages) of chest radiographs (CXRs) from each country by classification. Bangladesh (BAN), South Africa (SAF), Mali (MAL), Zambia (ZAM), Kenya (KEN), Thailand (THA), and Gambia (GAM).

The initial readers assessed between 532 and 657 images each and had a median accuracy of 67% (range 40%-74%). The arbitrators assessed between 1268 and 1274 images each and had median accuracy of 76% (range 59%-77%). The initial reviewers had 44% concordance, while the arbitrators had 49% concordance. The agreement between the first two initial readers increased with children's age ( Figure 1). Overall, 611(15%) of the CXR images had consolidation only, 993 (24%) had infiltrates only, 464 (11%) had both consolidation and infiltrates, 1692 (40%) were normal, and 409 (10%) were uninterpretable. The percentage of images that were considered uninterpretable in each site ranged between 4% and 20%. Normal CXR accounted for approximately half of the images in all sites except Zambia and South Africa (31% and 28%, respectively) ( Figure 2).

### Models

CXRs from 20% (802/4008) of patients were set aside for final model validation, while the rest were used for model training and validation. Convolutional neural networks were trained to classify the CXRs into one of the five WHO categories: consolidation; other infiltrate; both consolidation and other infiltrate; normal or uninterpretable. Model performance was assessed on the test data set using multi-class accuracy and area under the curve (AUC, one vs rest). The models were trained using Pytorch 1.7 running on a desktop with 32GB RAM and a single Nvidia Titan RTX graphical processing unit ( Paszke *et al*., 2019). The Python code for this analysis is available on Github. All libraries used in the analysis are open source and can be downloaded using Python package installer or from respective websites.

For simplicity, we used pre-trained ResNet18, ResNet34 and ResNet50 model architectures from the torchvision version 0.8.2 library for all our experiments ( Marcel & Rodriguez, 2010). The ResNet models' last fully connected layer was replaced with a fully connected layer with five output units – one for each WHO category.

### Incorporating individual reader annotations

**Figure 3.**
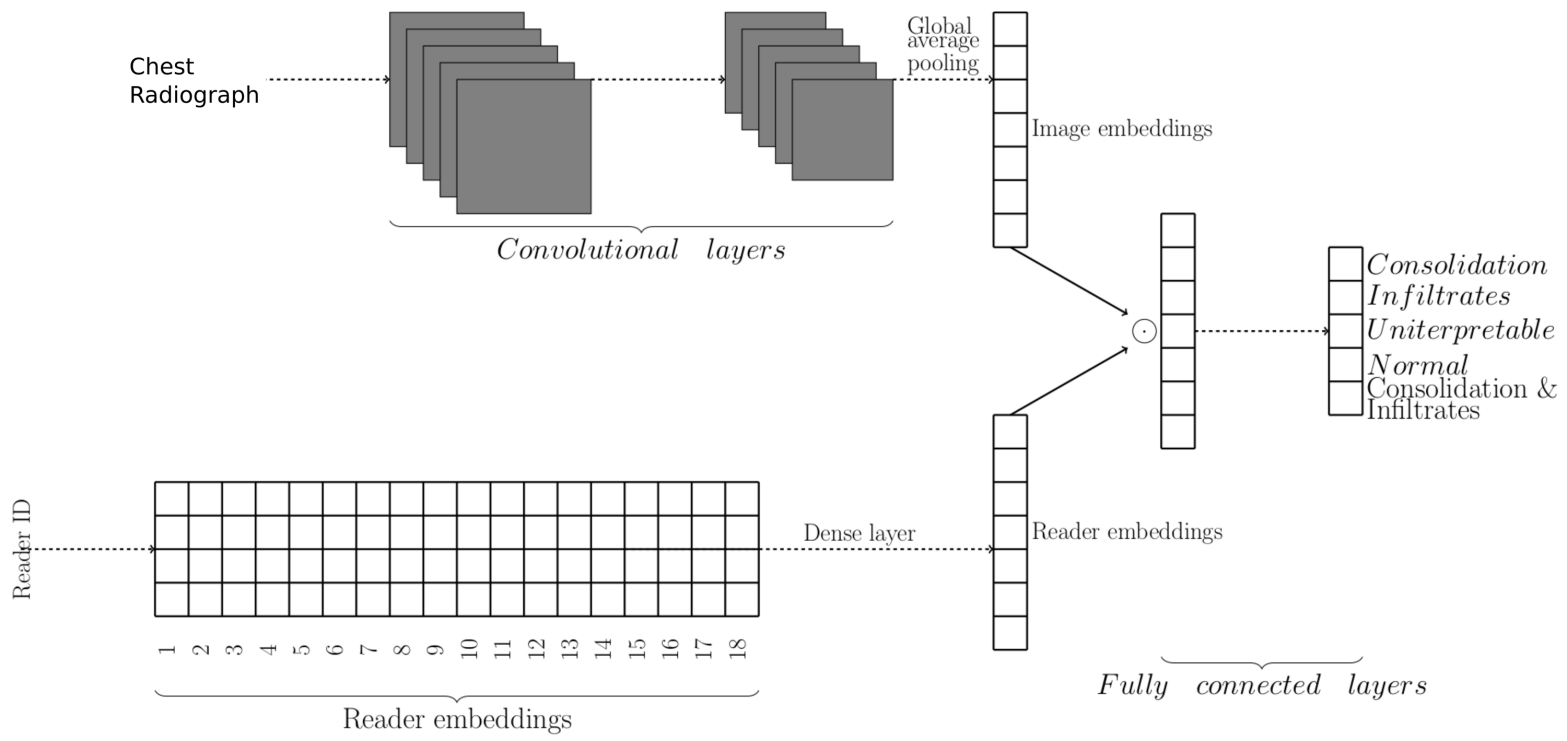
Model for classifying chest radiographs (CXRs) conditional on reader identity. The upper part of the network learns CXR embeddings, while the lower part learns reader embeddings. CXR and reader embedding are combined using element-wise multiplication. The reader embeddings allow the model to predict how each reader would classify a given image.

The ResNet models have a global average pooling (GAP) operation after the final convolutional layer. The output of GAP is passed to a single fully connected layer which outputs the model prediction. Consequently, we can consider the output of GAP as image embedding that act as input for a linear classifier (last fully connected layer). We extended the ResNet models to include reader embeddings by embedding reader identifiers into a vector of 32 units. A fully connected layer was then used to project the reader embedding to have the same dimension as image embedding. An identity, rectified linear unit (ReLU), hyperbolic tangent (tanh), or sigmoid activation was applied to the projected reader embeddings. Finally, element-wise multiplication was used to combine the reader and the image embeddings, and a fully connected layer with softmax activation was appended for prediction ( Figure 3).

We sampled one occurrence of each training CXR in every epoch so that models with and without embeddings had the same number of weight updates per epoch. In addition, we used each reader's annotation as labels during training, unlike in models without reader embeddings where the final classification was used. There were 18 readers in total. Thus, 18 predictions could be made for every CXR image. During inference, the 18 predictions were then aggregated to give the final prediction using an unweighted mean.

### Data pipeline and image augmentation

All CXR images were first down-sampled to 300x300 pixels to reduce the computation cost of training the models. Then, as with the original ResNet implementation, all models were trained on images of dimensions 3 x 224 x 224 ( He *et al*., 2015). The validation pipeline applied centre crop to resize the images to 224 x 224 pixels and applied normalization. The training pipeline resized the images to 224 x 224 pixels by applying random resized cropping. The training pipeline also applied random brightness and contract augmentation, random horizontal flip, and random affine transformations (rotation and sheer) to reduce overfitting. Finally, both validation and training pipelines applied normalization similar to ImageNet data set by subtracting (0.485, 0.456, 0.406) and dividing by (0.229, 0.224, 0.225) from the red, green, and blue channels.

### Hyper-parameter optimization

 We used the Asynchronous Successive Halving Algorithm (ASHA) to identify optimal hyper-parameters for all models using the raytune library in python ( Li *et al*., 2020; Liaw *et al*., 2018). We performed ASHA hyper-parameter search by randomly sampling 300 hyper-parameter configurations from the hyper-parameter search space and then stopping poor-performing configurations after 10, 20, 40, and 80 epochs. The hyper-parameters tuned for models without reader embeddings were training batch size, dropout proportion, weight decay coefficient for convolutional and fully connected layers, learning rate, the proportion of training images with affine transformation augmentation, and the proportion of training images with brightness and contrast adjustment augmentation. Models with reader embeddings had additional hyper-parameters for maximum L2-norm of reader embeddings, learning rate for embedding weights and weight decay coefficient for the fully connected layer that project reader embedding to have the same dimension as image embeddings. All models were trained for a maximum of 150 epochs, with the learning rate halved after 50 and 100 epochs.

## Results

**Figure 4.**
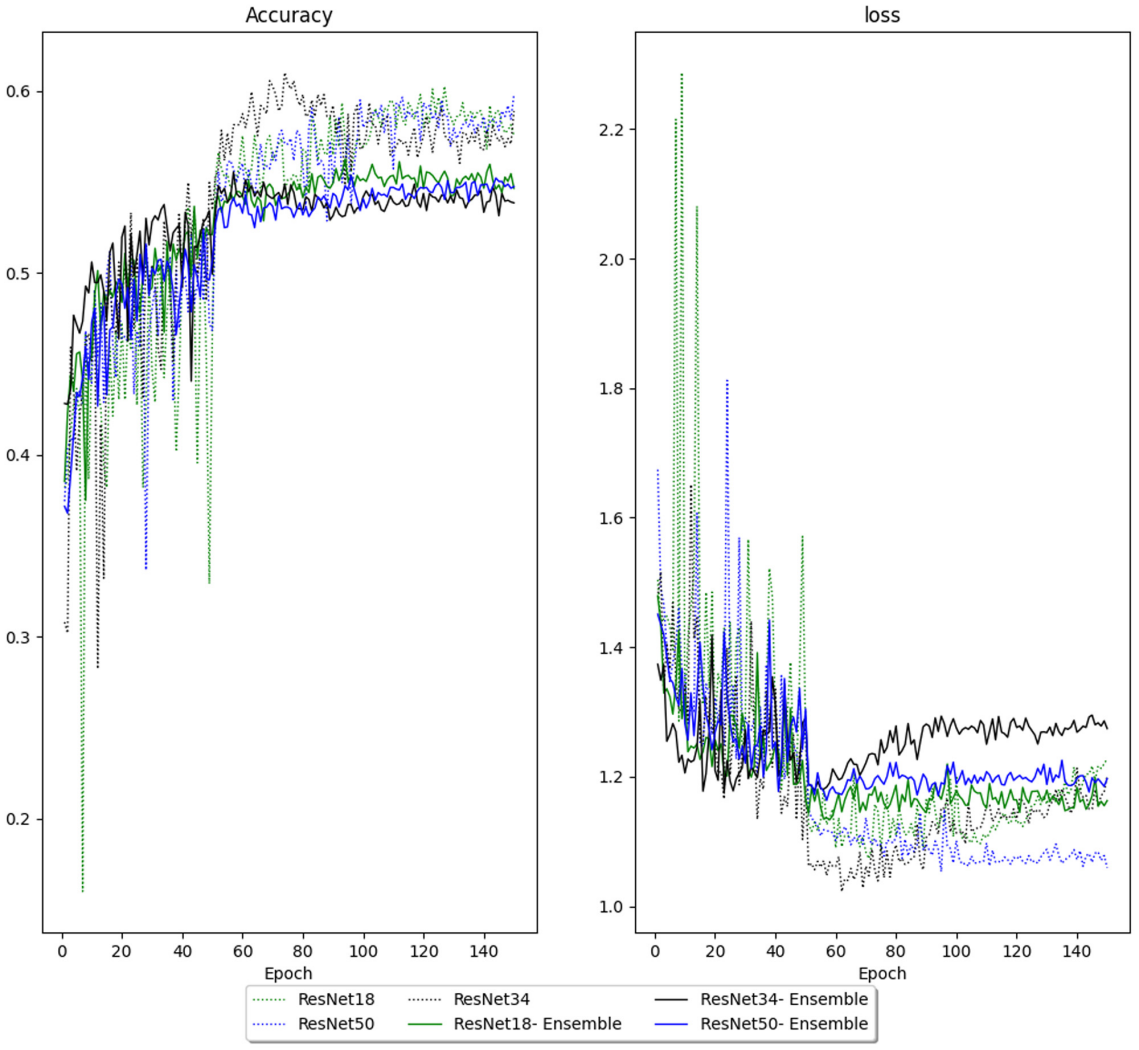
Validation loss and accuracy of models with and without reader embedding. For models with reader embeddings (ensemble), the target outcome is individual reader annotations instead of the final classification. The learning rate is annealed after 50 and 100 epochs.

Models with reader embedding were trained to predict how a given reader would classify an image instead of final/aggregate classification. During training, the models with reader embeddings had higher cross-entropy loss and lower accuracy on the validation data than models trained to predict the final classification ( Figure 4). However, models with reader embedding made 18 predictions for each CXR, which produced predictions with better accuracy and AUCs after aggregation. Reader embedding improved multi-class accuracy in ResNet18 (0.61 vs 0.59), ResNet34 (0.6 vs 0.57) and ResNet50 (0.6 vs 0.59). Models with reader embeddings also had higher unweighted mean AUC for ResNet18 (0.86 vs 0.84), ResNet34 (0.86 vs 0.82) and ResNet50 (0.86 vs 0.84). Disaggregated AUCs are shown in Table 1. Figure 4 shows that models without reader embedding had wider validation loss and accuracy fluctuations in the first 50 epochs of training (before the first learning rate reduction). Optimal hyper-parameters for each of the models are listed in Table 2.

**Table 1.** Area under the curve (AUC, one-vs-rest) and multi-class accuracy comparing models with and without reader embeddings. Bold figures denote the best AUC or accuracy for each model architecture. CXR = chest radiograph.

		AUC		Accuracy	
		without reader embeddings	with reader embeddings	without reader embeddings	with reader embeddings
**Model**	**CXR class**				
**ResNet18**	Consolidation	0.83	**0.84**	0.59	**0.61**
	Consolidation and other infiltrate	0.87	**0.9**		
	Normal	0.86	**0.88**		
	Other infiltrate	0.79	**0.81**		
	Uninterpretable	0.85	**0.88**		
**ResNet34**	Consolidation	0.79	**0.85**	0.57	**0.6**
	Consolidation and other infiltrate	0.84	**0.89**		
	Normal	0.86	**0.87**		
	Other infiltrate	0.78	**0.8**		
	Uninterpretable	0.83	**0.87**		
**ResNet50**	Consolidation	**0.84**	0.83	0.59	**0.6**
	Consolidation and other infiltrate	0.87	**0.89**		
	Normal	0.87	**0.88**		
	Other infiltrate	0.78	**0.81**		
	Uninterpretable	0.84	**0.88**		

**Table 2.** Optimal hyper-parameters for models with and without reader embeddings.

	Model					
	ResNet18		ResNet34		ResNet50	
	without reader embeddings	with reader embeddings	without reader embeddings	with reader embeddings	without reader embeddings	with reader embeddings
**Hyper-parameter**						
Activation function for projected reader embeddings		identity		identity		identity
Batch size	8	32	16	16	8	16
Dropout	0.22	0.28	0.35	0.05	0.36	0.01
L2 regularization of convolutional layers	0.199886	1.9E-05	0.000163	5E-06	0.256886	0.00443
L2 regularization of fully connected layer	4.8E-05	5.1E-05	0.000242	5E-06	1E-05	2.7E-05
L2 regularization of fully connected layer projecting the reader embeddings		4E-06		0.291381		2E-06
Learning rate for convolutional layers	2.1E-05	0.000346	0.000474	0.000282	2E-05	3.6E-05
Learning rate for fully connected layer	0.002909	0.049604	0.00163	0.023335	2.6E-05	0.029704
Learning rate for fully connected layer projecting the reader embeddings		0.000923		0.000141		0.020499
Learning rate for reader embeddings		0.001818		0.007738		0.009301
Max L2-norm of reader Embeddings		1		4		1
Proportion of images with color brightness and contrast augmentation	0.2	0.5	0	0	0.5	1
Proportion of training images with affine transformation augmentation	0.8	0.2	1	0.2	1	0.5

**Figure 5.**
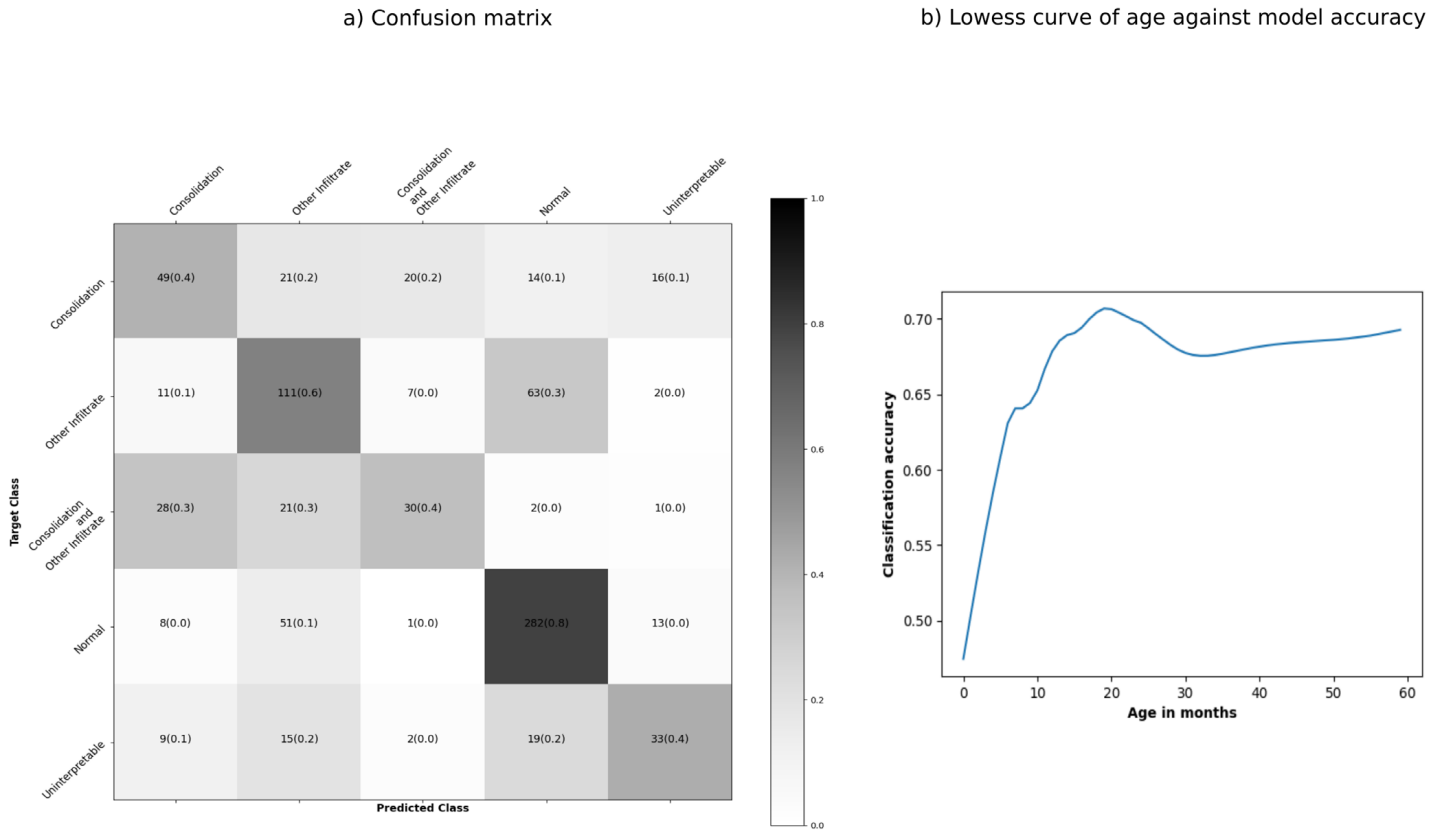
Confusion matrix and lowess curve of age against accuracy for the model with the highest accuracy. Tiles of the confusion matrix are shaded by the proportion of chest radiographs (CXRs) predicted to belong to each class (row proportions).

The best model had an accuracy of 61% and correctly classified 80% of normal CXR. For CXR with both consolidation and infiltrates, 30% were misclassified as consolidation only and 30% as infiltrates only. Thirty per cent of CXR with infiltrates were misclassified as normal ( Figure 5). There was wide variation in model accuracy across sites: Bangladesh (71%), Gambia (67%), Kenya (70%), Mali (59%), South Africa (53%), Thailand (65%) and Zambia (45%). The model had lower accuracy for children below 12 months of age than older children (58% vs 68%). Figure 5b shows that the prediction accuracy improved with children's age.

## Discussion

Models with reader embeddings were better at classifying CXR images regardless of model architecture (ResNet18, ResNet34 or ResNet50). The best model with reader embeddings had an accuracy of 61% compared to 59% in models ignoring individual reader classification, reflecting a 3.4% improvement. While some of the improvement in models with reader embeddings could be explained by the additional parameters, the cost of training was only slightly higher. Models with reader embeddings had more parameters: ResNet50 had 67,416 additional parameters while ResNet18 and ResNet34 had 16,928 additional parameters each. This increase in the number of parameters is minimal, considering that the models have tens of millions of parameters (less than 1% increment).

Individual reader annotations are more likely to be misclassified compared to labels obtained by aggregating all readers' annotations, which might make model training difficult ( Nettleton *et al*., 2010; Pechenizkiy *et al*., 2006). Consequently, models with reader embeddings had lower validation accuracy during training than models trained to predict the aggregated annotation. However, models with reader embedding made multiple predictions for each CXR (one prediction per reader) which after aggregation had higher accuracy compared to predictions from models predicting the final annotation. We used unweighted mean to aggregate predictions from models with reader embeddings which might not be optimal. A separate model can be trained to learn weights to assign to predictions from each reader in a manner similar to stacking ( Ozay & Vural, 2013; Wolpert, 1992).

The model with reader embedding was equivalent to the model without reader embeddings if all the values of reader embedding have value one (reader and image embedding were combined using element-wise multiplication). If we consider image embeddings as features extracted from a given image, the learned reader embedding allowed different readers to assign different weights to each image feature. The activation function applied to the reader embeddings determined whether the direction of association between image features and predicted class could be different for different readers. That is, for activation functions that don't output negative values (ReLU and sigmoid), the direction of association between a given image feature and the predicted class could not differ by reader.

The best model had lower accuracy than the initial readers (61% vs 67%). However, the comparison of model and readers accuracy was tilted in favour of readers because the readers' annotations were used to arrive at the final/aggregate annotation. Despite the modest performance in performing five-way classification, the model had high accuracy when identifying normal CXRs (80% accuracy). Therefore, the model might be useful in classifying normal vs abnormal CXRs. Studies comparing the performance of clinicians/radiologists and machine learning models on independent test data-sets have shown that models can outperform human readers. Rajpurkar developed models that achieved average radiologists' performance in detecting pneumonia and 13 other respiratory conditions (2017b; 2018). Furthermore, we trained the model using a relatively small data set, which might negatively affect model performance. Dunnmon found that increasing the number of CXR images from 2,000 to 20,000 increased AUC from 0.84 to 0.95 (2018).

The agreement between the two initial readers and model accuracy improved with children's age – both the readers and models had difficulties interpreting CXR from younger children. Difficulty in interpreting CXR from younger children by both the readers and models may be due to challenges obtaining quality CXR images from very young children. Machine learning models may also face challenges classifying CXR of smaller or/and younger children due to the presence of body parts besides the lungs (limbs and head). Therefore, we applied random cropping during model training to make the models robust to the presence of other body parts.

There was a wide variation in model accuracy among sites (range 45% to 71%) which may be explained by differences in pathology distribution. The model performance was poorest for Zambia and South Africa - the sites with the lowest proportion of normal images - because the model was better at classifying normal CXR than other pathologies. On the other hand, the model achieved an accuracy of 71% in Bangladesh despite the CXR in Matlab being acquired via analogue means, suggesting that the models can be applied in settings where digital CXR machines are not available.

Similar to other studies, all models were fitted using CXR images down-sampled to 224 by 224 pixels ( Dunnmon *et al*., 2018; Rajpurkar *et al*., 2017; Wang & Xia, 2018). While such down-sampling may hinder detection of certain pathologies such as infiltrates, training models using high-resolution CXRs is computationally costly and may not be feasible at scale.

Model performance was assessed using a single hold out test data set instead of K-fold cross-validation due to restriction in computation resources. While we believe the test set was large enough to assess model performance, K-fold cross-validation would have allowed computation of confidence intervals around model accuracy. Furthermore, slitting data set by site would allow assessing model generalizability to sites not included during model training. Assessing model generalizability across sites is important because factors such as differences in machines used to acquire CXR images and acquisition procedures may degrade model performance during implementation phases, hindering application of machine learning models in epidemiological studies carried out in multiple sites. Therefore, further work is required to determine how robust the models are to variations in CXR machines and radiographers preparing the images.

## Conclusion

In summary, we have demonstrated that machine learning models for CXR classification can benefit from incorporating individual reader's classification instead of directly predicting the final classification. Furthermore, machine learning models demonstrated here are unlikely to suffer from inter-reader and intra-reader because they are deterministic. Consequently, the models might be suitable for multisite studies or studies conducted over a long time.

## Data availability

### Underlying data

Data will be made publicly available in ClinEpiDB. Investigators can submit a data request describing the purpose for which the data will be used which will be shared and reviewed by the PERCH Executive Committee prior to approval. ( Fancourt *et al*., 2017a).

### Extended data

Analysis code available from: https://github.com/pmwaniki/xray-analysis.

Archived analysis code as at time of publication: https://doi.org/10.5281/zenodo.5501796 ( Mwaniki, 2021).

License: MIT license.
