# Supplementary material for: Using Machine Learning Methods Incorporating Individual Reader Annotations to Classify Paediatric Chest Radiographs in Epidemiological Studies
Source: Wellcome Open Res. 2022 Aug 25;6:309. Originally published 2021 Nov 12. [Version 2] doi: 10.12688/wellcomeopenres.17164.2 (PMC9463539; doi:10.12688/wellcomeopenres.17164.2)
Supplement: Peer review history

# Peer review: Reviewer response for version 2

**DOI:** 10.21956/wellcomeopenres.19955.r52168

**Published:** 2022-09-09

**Authors:** Rendani Mbuvha

**Affiliations:** 1 School of Statistics and Actuarial Science, University of Witwatersrand, Johannesburg, South Africa; 2 University College London, London, UK; 3 Queen Mary University of London, London, UK

All requested changes have been made.

Is the work clearly and accurately presented and does it cite the current literature?

Yes

If applicable, is the statistical analysis and its interpretation appropriate?

Yes

Are all the source data underlying the results available to ensure full reproducibility?

Yes

Is the study design appropriate and is the work technically sound?

Yes

Are the conclusions drawn adequately supported by the results?

Yes

Are sufficient details of methods and analysis provided to allow replication by others?

Partly

Reviewer Expertise:

Machine Learning, Bayesian Inference

I confirm that I have read this submission and believe that I have an appropriate level of expertise to confirm that it is of an acceptable scientific standard.

# Peer review: Reviewer response for version 2

**DOI:** 10.21956/wellcomeopenres.19955.r52169

**Published:** 2022-09-01

**Authors:** Sameer Antani

**Affiliations:** 1 National Library of Medicine, National Institutes of Health, Bethesda, MD, USA

The authors have adequately addressed the concerns raised in the earlier review.

Is the work clearly and accurately presented and does it cite the current literature?

Partly

If applicable, is the statistical analysis and its interpretation appropriate?

Yes

Are all the source data underlying the results available to ensure full reproducibility?

No

Is the study design appropriate and is the work technically sound?

Yes

Are the conclusions drawn adequately supported by the results?

Yes

Are sufficient details of methods and analysis provided to allow replication by others?

Yes

Reviewer Expertise:

Machine Learning, Artificial Intelligence, Medical Imaging, Computer-Aided Diagnostics

I confirm that I have read this submission and believe that I have an appropriate level of expertise to confirm that it is of an acceptable scientific standard.

# Peer review: Reviewer response for version 1

**DOI:** 10.21956/wellcomeopenres.18958.r48877

**Published:** 2022-03-15

**Authors:** Sameer Antani

**Affiliations:** 1 National Library of Medicine, National Institutes of Health, Bethesda, MD, USA

The authors aim to use Machine Learning models to standardize CXR analysis across studies at various centers with an aim to reduce inter- and intra-reader variability. Each model was trained to individual reader and an unweighted mean was used to aggregate the predictions. 

 Overall the work is well done and important toward producing a portable algorithm that reduces the variability in human interpretation. 

 Questions/Concerns:

1. The authors state that 20% of the patients were set aside for model testing (i.e. verification/validation) and remaining were used for training and validation. It is recommended that the authors use testing instead of validation for clarity. Also, how were the 20% selected? Did they represent all datasets uniformly?
2. All images were acquired, save for 2 for which they were scanned. Was the same scanner manufacturer used? Were the settings identical? ML models have shown sensitivity to scanners and settings. Was this addressed? How?
3. No visualization studies have been presented? Did the authors verify that the models were paying attention to the right areas? Recent works by Rajaraman, *et al* ( https://www.frontiersin.org/articles/10.3389/fgene.2022.864724/full) have shown that models can result in high accuracy even when they are not using the lung field to arrive at the prediction.

Is the work clearly and accurately presented and does it cite the current literature?

Partly

If applicable, is the statistical analysis and its interpretation appropriate?

Yes

Are all the source data underlying the results available to ensure full reproducibility?

No

Is the study design appropriate and is the work technically sound?

Yes

Are the conclusions drawn adequately supported by the results?

Yes

Are sufficient details of methods and analysis provided to allow replication by others?

Yes

Reviewer Expertise:

Machine Learning, Artificial Intelligence, Medical Imaging, Computer-Aided Diagnostics

I confirm that I have read this submission and believe that I have an appropriate level of expertise to confirm that it is of an acceptable scientific standard, however I have significant reservations, as outlined above.

# Response

**Published:** 2022-08-18

**Authors:** Paul Mwaniki

**Affiliations:** KEMRI - Wellcome Trust Research Programme, Kenya

1. The authors state that 20% of the patients were set aside for model testing (i.e. verification/validation) and remaining were used for training and validation. It is recommended that the authors use testing instead of validation for clarity. Also, how were the 20% selected? Did they represent all datasets uniformly?

**Response:** Thank you for pointing out that we have used the term ‘validation’ instead of ‘testing’. We have also indicated that we randomly selected data from 20% of patients for the final model. We modified the text to clarify: “A random sample of CXRs from 20% (802/4008) of patients from all sites were set aside for final model evaluation/testing, while the rest were used for model training and hyper-parameter selection. Simple random sampling was used to select CXRs to be included in the testing data set such that all patients had an equal chance of being selected.”

   

 2. All images were acquired, save for 2 for which they were scanned. Was the same scanner manufacturer used? Were the settings identical? ML models have shown sensitivity to scanners and settings. Was this addressed? How?

**Response:** We have now stated that different scanners were used at different sites. While data augmentation may lead to models that are robust to differences in CXR machines and scanners, the single train/test split employed in this study could not allow such an assessment. 

 3. No visualization studies have been presented? Did the authors verify that the models were paying attention to the right areas? Recent works by Rajaraman, et al (https://www.frontiersin.org/articles/10.3389/fgene.2022.864724/full) have shown that models can result in high accuracy even when they are not using the lung field to arrive at the prediction.

**Response:** Thank you for suggesting visualizations that enhance the interpretability of model predictions. We have added a Grad-CAM visualization that shows that the model used relevant regions on chest x-rays in making the predictions.

# Peer review: Reviewer response for version 1

**DOI:** 10.21956/wellcomeopenres.18958.r48109

**Published:** 2022-02-04

**Authors:** Rendani Mbuvha

**Affiliations:** 1 School of Statistics and Actuarial Science, University of Witwatersrand, Johannesburg, South Africa; 2 University College London, London, UK; 3 Queen Mary University of London, London, UK

The manuscript addresses inter and intra-reader variability when reading chest radiographs (CXR). The authors employ convolutional neural networks which are fused with reader annotations. The novel idea of the work lies in combining image and text embeddings (of reader annotations) as features. The results show that including this addition of text embeddings as features does improve predictive performance. This work is a meaningful contribution in areas where timely and low-resource CXR interpretation/classification is required.

 Some questions and comments for the authors:

- Authors should explain/make clear to the reader how text embeddings were generated - this is my primary identified weakness of the manuscript.
- Authors refer to the fully connected layer classifier as a linear classifier (bottom of page 4) - this is not entirely correct as the activation functions used are still non-linear. 
- On page 5, the authors refer to "contract augmentation" - is it supposed to read as "contrast augmentation" rather.

Is the work clearly and accurately presented and does it cite the current literature?

Yes

If applicable, is the statistical analysis and its interpretation appropriate?

Yes

Are all the source data underlying the results available to ensure full reproducibility?

Yes

Is the study design appropriate and is the work technically sound?

Yes

Are the conclusions drawn adequately supported by the results?

Yes

Are sufficient details of methods and analysis provided to allow replication by others?

Partly

Reviewer Expertise:

Machine Learning, Bayesian Inference

I confirm that I have read this submission and believe that I have an appropriate level of expertise to confirm that it is of an acceptable scientific standard, however I have significant reservations, as outlined above.

# Response

**Published:** 2022-08-18

**Authors:** Paul Mwaniki

**Affiliations:** KEMRI - Wellcome Trust Research Programme, Kenya

1. Authors should explain/make clear to the reader how text embeddings were generated - this is my primary identified weakness of the manuscript.

**Response: **Thank you for pointing out that the procedure of creating reader embeddings is not clear. No text embeddings were used. Instead, we performed entity embeddings of categorical variables for reader identifiers instead of one hot encoding. We have added the following text to make it clear: “We extended the ResNet models to include reader embeddings which transformed each reader’s identifier into a vector of 32 units using entity embeddings for categorical variables (Guo and Berkhahn 2016).”

 2. Authors refer to the fully connected layer classifier as a linear classifier (bottom of page 4) - this is not entirely correct as the activation functions used are still non-linear. 

**Response: **While a softmax activation function was applied, the last layer consists of a single fully connected layer (no hidden layers). Therefore, the outputs of the last fully connected layer are a linear combination of the inputs. To avoid confusion, we have removed the phrase “linear classifier” and modified the sentence to read: ”Consequently, we can consider the output of GAP as image embeddings that act as input for the last fully connected layer.” 

 3. On page 5, the authors refer to "contract augmentation" - is it supposed to read as "contrast augmentation" rather.

**Response: **Thank you for pointing out the spelling mistake. The sentence has now been corrected to read “contrast augmentation”.
